# Progestin-primed milder stimulation with clomiphene citrate yields fewer oocytes and suboptimal pregnancy outcomes compared with the standard progestin-primed ovarian stimulation in infertile women with polycystic ovarian syndrome

**DOI:** 10.1186/s12958-018-0373-7

**Published:** 2018-05-28

**Authors:** Hongjuan Ye, Hui Tian, Wen He, Qifeng Lyu, Yanping Kuang, Qiuju Chen, Lihua Sun

**Affiliations:** 1 Centre of assisted reproduction, Shanghai East Hospital, Tongji University, Shanghai, People’s Republic of China; 2 Department of Assisted Reproduction, Shanghai Ninth People’s Hospital, Shanghai Jiaotong University School of Medicine, Shanghai, People’s Republic of China

**Keywords:** Polycystic ovarian syndrome, Progestin-primed ovarian stimulation, Clomiphene citrate, In vitro fertilization, Freeze-only

## Abstract

**Background:**

Oral progestin has recently been used to prevent premature LH surges in ovarian stimulation, and this progestin-primed ovarian stimulation (PPOS) is effective and safe in patients with different ovarian reserves. The current data are lacking regarding how to individualize the gonadotropin dose and regimen for women with polycystic ovarian syndrome (PCOS). A retrospective cohort trial was performed to evaluate the efficacy of progestin-primed milder stimulation with clomiphene citrate (CC) compared to the standard progestin-primed ovarian stimulation (PPOS) protocol for infertile women with PCOS.

**Methods:**

A total of 220 PCOS women were collected and classified into the study group (HMG 150 IU/d + CC 50 mg/d + MPA 10 mg/d) and control group (HMG 225 IU/d + MPA 10 mg/d). Ovulation was triggered by GnRH agonist 0.1 mg and hCG 1000 IU when dominant follicles matured. Viable embryos were cryopreserved for later transfer. The primary endpoint was the ongoing pregnancy rate. Secondary outcomes included the cycle characteristics and the live birth rate.

**Result(s):**

The study group consumed less HMG (1470.0 ± 360.1 IU vs 1943.8 ± 372.0 IU, *P* < 0.001) and harvested fewer oocytes than the control group (12.2 ± 7.4 vs 18.2 ± 9.7, *P* < 0.001). The study group showed a higher mid-follicular LH concentration (4.49 ± 2.49 mIU/ml vs 2.52 ± 2.09 mIU/ml, *P* < 0.05) but no endogenous LH surge. No between-group difference was found in the incidence of ovarian hyperstimulation syndrome (OHSS) (0.91% vs 0.91%, *P* > 0.05). The cumulative ongoing pregnancy rate and live birth rate per patient were lower but did not reach significance compared with the control group (71.8% vs 81.8 and 64.5% vs 75.5%, respectively, both *P* > 0.05).

**Conclusion(s):**

The milder PPOS with CC in PCOS women led to lower oocyte yields and suboptimal pregnancy outcomes compared to the standard PPOS treatment. The two regimens both achieved a low incidence of OHSS. The results from the CC combination regimen provide a new insight for developing a more patient-friendly protocol for PCOS women.

## Background

Polycystic ovarian syndrome (PCOS) is an endocrine disorder affecting 5–10% of reproductive-age women worldwide [1]. Approximately 74% of women with PCOS seeks pregnancy assistance, including induced ovulation, insemination or in vitro fertilization (IVF) [2]. However, PCOS women undergoing IVF treatment typically produce an increased number of oocytes, which are often of poor quality, leading to a lower fertilization rate and a higher miscarriage rate [3]. They also face a higher risk of moderate/severe ovarian hyperstimulation syndrome (OHSS) [4, 5].

Thanks to the progress of vitrification, oral progestin has been successfully used to prevent premature LH surges in women undergoing ovarian stimulation [6–8]. This progestin-primed ovarian stimulation (PPOS) yields a comparable pregnancy outcome, although it consumes a slightly higher gonadotropin dosage than conventional short protocols [6]. PPOS is approved its efficacy and safety in the population of low-ovarian-reserve, normal-ovarian-reserve and PCOS women [8–10], so PPOS in combination with a freeze-only policy shows good potential to compete with conventional protocols. The existing data from clinical trials often use the equal initiating doses of gonadotropin for women with or without PCOS, but relevant data are lacking about how to individualize the gonadotropin dose and regimen for PCOS women undergoing ovarian stimulation.

Clomiphene citrate (CC) has been a first-line drug for ovulation induction for anovulatory infertility. Its advantages include its oral route, low costs and easy access compared to gonadotropins [11, 12]. CC is also used in GnRH antagonist protocols, and the combination of CC and GnRH antagonist is likely to reduce the risk of OHSS, medication costs and gonadotropin duration compared to those without CC, but it accompanies with an increased risk of premature LH surges [13–15]. Limited data are available about the role of CC in PPOS for PCOS women [16]. In this trial, we attempted to test the hypothesis that in women with PCOS, the milder stimulation of PPOS in combination with CC would provide an acceptable clinical outcome compared with standard PPOS protocol using conventional initiating dose of gonadotropin. Our ultimate aim was to optimize the PPOS protocol and make it more patient-friendly.

## Methods

### Study setting and subjects

**Fig. 1:**
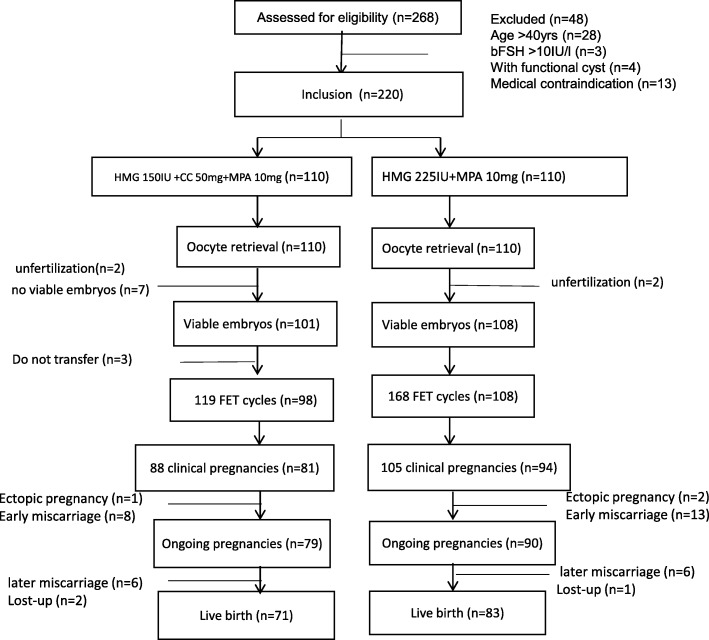
Study flowchart

A retrospective cohort trial was conducted at the department of assisted reproduction of the Ninth People’s Hospital of Shanghai Jiaotong University School of Medicine. This study was approval by the Ethics Committee (Institutional Review Board) of Shanghai Ninth People’s Hospital. The PCOS diagnosis criteria followed the Rotterdam consensus. PCOS was diagnosed by the presence of menstrual disturbance combined with either hyperandrogenism (hirsutism or hyperandrogenaemia) or polycystic ovary on ultrasonography (defined as an ovary that contained ≥12 antral follicles) and excluded other causes of hyperandrogenism (congenital adrenal hyperplasia, Cushing’s syndrome, androgen-producing tumours) and ovulation dysfunction (hyperprolactinaemia and thyroid dysfunction). In addition, this study only included women no more than 40 years of age and with baseline serum FSH no more than 10 mIU/ml. Women with functional cysts on the ovaries or medical conditions that contraindicated assisted reproductive technology and/or pregnancy were excluded. A total of 220 infertile women with PCOS from April 2014 to November 2015 were included and classified into the study group (HMG + MPA + CC) and the control group (HMG + MPA). A flowchart of the study is shown in Fig. 1.

### Ovarian stimulation protocol

In the study group, a low dose of HMG (150 IU daily), CC 50 mg and MPA 10 mg daily were started from cycle day 3 or after an episode of withdrawal bleeding. MPA was used to prevent premature ovulation during the ovarian stimulation. Follicle monitoring by transvaginal ultrasound and serum hormone measurements (FSH, LH, E_2_ and progesterone) were performed 5 days later. HMG doses were then adjusted according to the ovarian response (range 150–300 IU daily). Oocyte maturation was triggered by triptorelin 0.1 mg (Decapeptyl, Ferring Pharmaceuticals, Germany) and urinary human chorionic gonadotropin (hCG 1000 IU, Lizhu Pharmaceutical Trading Co., China) when at least three follicles reached diameters of 18 mm or more. Cumulus oocyte complexes were collected 36 h later. All follicles larger than 10 mm in diameter were aspirated.

In the control group, HMG 225 IU and MPA 10 mg daily were initiated from cycle day 3 or after an episode of withdrawal bleeding. Follicle monitor and hormone assay were performed 5 days later. HMG dose was then adjusted according to the ovarian response, and MPA dose was consistent up to the trigger day. The criteria of mature follicle and the trigger methods were the same as above.

Fertilization was carried out in vitro after oocyte retrieval depending on the semen parameters and previous fertilization situation. Embryos were examined for the number or regularity of blastomeres and the degree of fragmentation. All top-quality cleavage-stage embryos (grade 1 and grade 2, 6-cell embryos and above) were frozen within three days after oocyte retrieval. The non-top-quality embryos were placed in further extended culture, and good-morphology blastocysts were frozen. Cleavage-stage embryos and blastocysts were frozen by vitrification as described previously [17].

### Endometrium preparation and FET

Endometrium preparation for FET was arranged on the second cycle after oocyte retrieval. The first choice was using a letrozole-induced- ovulation cycle. Letrozole 5 mg was administered for 5 days, and then, follicle growth was monitored beginning on day 10. At times, a low dose of HMG (75 IU/day) was used to stimulate follicle growth and endometrial lining. The timing of FET was performed 4 or 5 days later, after a spontaneous or hCG-induced LH surge. Hormone replacement treatment was recommended for patients with thin endometrium and patients in whom letrozole failed. Oral ethinyl oestradiol 75 mcg/day was administered from cycle day 3 onwards. Once the endometrial lining was > 8 mm thick, femoston (Solvay Pharmaceuticals B.V.) 8 mg/day was started. The time of thawing and transfer was determined on the third day after femoston administration [17]. Each patient received no more than two embryos at one time. Once pregnancy was achieved, the luteal support was continued until 10 weeks of gestation.

### Hormone measurement

Serum FSH, LH, E_2_, and progesterone were measured on menstrual cycle day 3, day 8–11 (after 5–7 days of stimulation), the trigger day and the day after trigger. Hormone levels were determined with chemiluminescence (Abbott Biological B.V. Netherlands). The lower limits of sensitivity were as follow: FSH 0.06 mIU/ml, LH 0.09 mIU/ml, E_2_ 10 pg/ml and progesterone 0.1 ng/ml. The upper limit of E_2_ measurement was 5000 pg/ml. If serum E_2_ on the trigger day or the day after was higher than the upper limit, it was recorded as 5000 pg/ml.

### Outcome variables

The primary measure analysed was the cumulative ongoing pregnancy rate, which was defined as the proportion of patients with ongoing pregnancy after the gestation age of 12 weeks. The secondary measures included the stimulation duration, gonadotropin consumption, incidence of premature LH surge and OHSS, the number of oocytes retrieved, the number of viable embryos, the proportion of mature oocytes and the live birth rate. The implantation rate was calculated as the number of gestational sacs visualized on transvaginal ultrasound divided by the number of transferred embryos. Clinical pregnancy was defined as the presence of foetal cardiac activity confirmed by transvaginal ultrasound. The cumulative live birth rate was defined as the total number of live births divided by all participants.

### Statistical analysis

The data were evaluated by Student’s t-test for continuous variables of normal distribution, the Mann-Whitney U-test for continuous variables of non-normal distribution, the x^2^-test or Fisher’s exact for categorical variables, as appropriate. All tests were two-sided, and *P* < .05 was considered statistically significant. All data were analysed using the Statistical Package for the Social Sciences for Windows (SPSS, version 19).

## Results

### Patient characteristics

The basal demographical and hormonal characteristics are shown in Table 1. A total of 220 patients completed this trial. There were no significant between-group differences in age, body mass index (BMI), previous IVF failures, infertility duration, menstrual cycle, indication for IVF or basal hormonal profile. All women completed one oocyte retrieval cycle, 208 patients had 1–15 viable embryos harvested, and 12 cases were cancelled before transfer due to either non-fertilization or no transferrable embryos. A total of 205 women completed 287 FET cycles in the following two years.

**Table 1:** The basic characteristics of PCOS women in this trial

	Study group (HMG + MPA + CC; *n* = 110)	Control group (HMG + MPA; *n* = 110)
Age (y)	30.5 ± 3.7	30.6 ± 3.4
Duration of infertility (y),	3.5 ± 2.6	3.9 ± 2.3
BMI (*kg*/*m*^2^) n (%)		
19~ 24.9	80(72.7%)	81(73.6%)
25~ 29.9	25(22.7%)	24(21.8%)
> =30	5(4.5%)	5(4.5%)
Previous IVF failures, n(%)		
0	89(80.9%)	88(80.8%)
1–3	21(19.1%)	22(19.2%)
Indication for IVF n (%)		
PCOS only	27(24.5%)	30(27.3%)
PCOS+ male factor	21(19.1%)	25(22.7%)
PCOS+ tubal factor	52(52.7%)	49(44.5%)
PCOS+ other	4(3.6%)	6(5.5%)
Menstrual cycle n (%)		
Regular	3(2.1%)	3(2.7%)
Oligomenorrhea	82(74.5%)	91(82.7%)
Amenorrhea	25(22.7%)	16(14.5%)
Antral follicle counts	17.9 ± 6.3	18.8 ± 7.1
Baseline hormones		
FSH (mIU/ml)	4.94 ± 1.10	5.0 ± 1.17
LH (mIU/ml)	5.03 ± 3.18	5.61 ± 3.36
E_2_(pg/ml)	32.54 ± 10.34	30.32 ± 12.52
P(ng/ml)	0.26 ± 0.19	0.27 ± 0.19
T (ng/ml)	0.35 ± 0.13	0.40 ± 0.19

No significant difference was found between the two groups (*P* > 0.05)

### Ovarian stimulation, follicle development, and oocyte performance

Clinical and cycle characteristics of ovarian stimulation in both groups are shown in Table 2. The study group (HMG + MPA + CC protocol) had a similar stimulation duration (9.2 ± 1.3 days vs 9.1 ± 1.2 days, *P* > 0.05) and consumed less HMG (1470.0 ± 360.1 IU vs 1943.8 ± 372.0 IU, *P* < 0.05). The numbers of oocytes retrieved, MII oocytes, and fertilized oocytes in the study group were significantly lower than those in the control group (*P* < 0.05). Consequently, the number of viable embryos in the study group was significantly lower than in the control group (4.8 ± 3.5 vs. 6.2 ± 3.7, *P* < 0.05). No between-group differences were found in the oocyte retrieval rate, but the maturation rate and the proportion of viable embryos per oocyte retrieved were better in the study group (respectively, 87.4% vs 80.3 and 39.5% vs 34.0%, both *P* < 0.05). The cycle cancellation due to zero viable embryos was significantly higher in the study group (9.1% vs 1.8%, *P* < 0.05). One patient experienced moderate or severe OHSS in each group (*P* > 0.05).

**Table 2:** The cycle characteristics of controlled ovarian stimulation in the two groups

	Study group (HMG + MPA + CC; *n* = 110)	Control group (HMG + MPA; *n* = 110)	*P* value
hMG doses (IU)	1470.0 ± 360.1	1943.8 ± 372.0	< 0.001
hMG duration (days)	9.2 ± 1.3	9.1 ± 1.2	0.594
No. of > 10 mm follicles on trigger day	17.6 ± 8.4	21.2 ± 8.3	0.001
No. of > 14 mm follicles on trigger day	13.8 ± 8.4	17.2 ± 9.3	0.004
No. of oocytes retrieved(n)	12.2 ± 7.4	18.2 ± 9.7	< 0.001
No. of maturation oocytes (n)	10.7 ± 6.3	14.6 ± 8.2	< 0.001
No. of fertilization (n)	8.6 ± 5.6	12.6 ± 7.7	< 0.001
No. of viable embryos(n)	4.8 ± 3.5	6.2 ± 3.7	0.007
Oocyte retrieval rate (%)	58.8% (1343/2285)	60.1% (2006/3335)	0.302
Oocyte maturation rate (%)	87.4% (1174/1343)	80.3% (1610/2006)	< 0.001
The proportion of viable embryo per oocyte retrieved (%)	39.5% (531/1343)	33.8% (678/2006)	0.001
Cancellation for no viable embryos (%)	9.1% (10/110)	1.8% (2/110)	0.018
Incidence of moderate/severe OHSS (%)	0.91% (1/110)	0.91% (1/110)	1.00

### Hormone profile during treatment

**Fig. 2:**
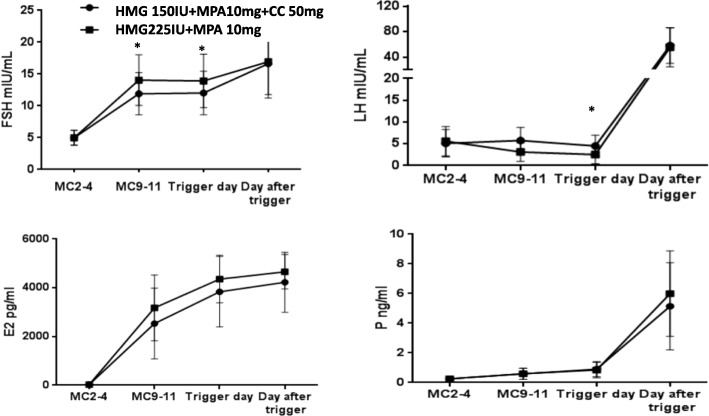
The dynamic changes in hormones during ovarian stimulation in the two groups

The serum concentrations of FSH, LH, E_2_ and P in the two groups are presented in Fig. 2. FSH in the study group was slightly lower than in the control group during the mid-follicular phase (*P* < 0.05). LH gradually decreased during ovarian stimulation in the control group; in contrast, LH in the study group showed a slight rise initially, followed by a downward trend, and the mean LH value on the trigger day was significantly higher than in the control group (4.49 ± 2.49 mIU/ml vs 2.52 ± 2.09 mIU/ml, *P* < 0.05). No endogenous LH surge occurred in either group (*P* > 0.05). The LH value on the post-trigger day showed a dramatic increase in the two groups, with no between-group difference (*P* > 0.05).

E_2_ increased gradually, accompanied by with multiple growing follicles during the ovarian stimulation, and no difference was found between the two groups (*P* > 0.05). The measured E_2_ values were underestimated in 114 cases due to the upper limit of 5000 pg/ml, so the comparison of E_2_ between the two groups was compromised. Serum P showed a gradual increase during ovarian stimulation and increased significantly after trigger in both groups.

### Pregnancy outcomes in FET cycles

The pregnancy outcomes from FET are shown in Table 3. A total of 287 FET cycles were completed in the two groups, including 66 women who finished at least two transfers. The control group yielded more embryos, which were able to finish more FET cycles in the following two years. The mean transfer cycles per patient were 1.5 in the control group and 1.1 in the study group in the following two years. A total of 560 embryos were thawed and the survival rate was 99.3% (556/560). The remnant embryos were, respectively, 350 and 303 in the control and study group, which were the suplus embryos in pregnant cases except for three cases in study group without their transfer.

**Table 3:** Pregnancy and live birth outcomes after FET

	Study group (HMG/MPA/CC)	Control group (HMG/MPA)	Risk Ratio (95% CI)	*P* value
Rates per embryo transfer				
Clinical pregnancy rate	73.9%(88/119)	62.5%(105/168)	1.70 (1.02, 2.85)	0.042
Implantation rate	52.2%(119/228)	42.7%(140/328)	1.47(1.04, 2.06)	0.027
Ectopic pregnancy rate	1.1%(1/88)	1.9%(2/105)	0.59(0.05, 6.64)	0.667
Miscarriage rate				
Early miscarriage	9.1%(8/88)	12.4%(13/105)	0.71(0.28,1.79)	0.465
Later miscarriage	6.8%(6/88)	5.7%(6/105)	1.21(0.38,3.89)	0.752
Ongoing pregnancy rate	66.4%(79/119)	53.6%(90/168)	1.71(1.05,2.78)	0.030
Live-birth rate	59.7%(71/119)^a^	49.4%(83/168)^a^	1.52(0.94,2.44)	0.086
Rates per participant				
Ongoing pregnancy rate	71.8%(79/110)	81.8%(90/110)	0.57(0.30,1.07)	0.079
Live birth rate	64.5%(71/110)^a^	75.5%(83/110)^a^	0.59(0.33,1.06)	0.077
Newborns				
Single birth (n)	50	60		
Single birthweight (g)	3270.4 ± 644.9	3357.0 ± 437.1		0.422
Twin birth (n)	21	23		
Twin birthweight (g)	2371.7 ± 460.7	2460.0 ± 423.5		0.497

^a^3 pregnant women lost to follow up to live birth (2 in study group and 1 in control group)

The synchronization methods of endometrium and embryo were similar between the two groups. The ongoing pregnancy rate per transfer and the implantation rate were significantly higher in the study group (respectively, 66.4% vs 53.6%; 52.2% vs 42.7%, *P* < 0.05) but the live birth rate per transfer was comparable between two groups (59.7% vs 49.4%, *P* > 0.05). Sixty-four women experienced twin pregnancies, including 6 women with vanishing syndrome. One triplet pregnancy occurred in each group, and both resulted in live births after operation of multifetal reduction. The proportions of multiple pregnancies, miscarriage and ectopic pregnancy were similar between groups (*P* > 0.05). The cumulative ongoing pregnancy and live birth rate per patient were lower in the study group but did not reach the significant difference (respectively 71.8% vs 81.8%; 64.5% vs. 75.5%; *P* > 0.05).

All newborns were examined with no congenital malformation except that oesophageal atresia was found in one baby of the control group and ventricular septal defect in one of the twin babies of the study group.

## Discussion

Milder stimulation, with its advantages of patient-friendliness, is a good solution for producing an acceptable pregnancy outcome and eliminating OHSS for high responders. This retrospective cohort trial demonstrated that the milder PPOS with CC led to lower oocyte yields and suboptimal pregnancy outcomes compared to the standard PPOS protocol in PCOS women, and the incidence of OHSS was low in both groups (0.91%).

In contrast to the standard protocol of HMG/MPA, the combination protocol of HMG/CC/MPA showed the characteristics of milder stimulation, such as fewer oocytes, fewer embryos, and a higher cancellation rate for non-transferrable embryos, but the harvested embryos showed good developmental potential in terms of implantation rate. This protocol led to fewer oocytes at the cost of low gonadotropin consumption. Serum FSH in mid-follicular phase was slightly lower in the CC combination protocol. The extent of ovarian stimulation may be regulated by using low dose of gonadotropin and CC, which leaves much flexibility for controlled ovarian stimulation. Although the proportion of viable embryos per retrieved oocyte was better in the CC combination protocol (39.5% vs 33.8%), the number of viable embryos was less than the 1.5 embryos from the standard protocol. The total number of transferrable embryos originating from the CC group was significantly lower, meaning the milder CC combination stimulation yielded suboptimal pregnancy outcomes compared with the standard protocol. But these results from a CC combination regimen may provide a new insight for develop a more patient-friendly protocol for PCOS women.

One of the strengths of this trial is that it verified the feasibility of CC co-administration in the PPOS protocol in PCOS women. Our results showed that the endogenous LH was well-suppressed during ovarian stimulation, and no spontaneous LH surge occurred in either group. CC increased endogenous gonadotropin secretion by blocking oestrogen’s negative feedback mechanism [11], as evidenced by a relatively higher LH level during the stimulation with CC in this trial. More important, no spontaneous LH surge occurred even with the relatively higher LH, which indicated that P’s suppression of pituitary function was still dominant. This phenomenon has also occurred in normo-ovulatory women using a CC combination protocol of PPOS [18]. These data indicate that, although CC and progesterone have separate action sites and pathways, the changed LH trend was the result of their collaborative action, so the two drugs may act independently and have the possibility to act collaboratively.

In PCOS women, multiple follicle growth in controlled ovarian stimulation leads to a higher risk of OHSS due to the higher sensibility and exaggerated response to gonadotropins. The incidence of moderate or severe OHSS in PCOS women is approximately 3.0 to 8.0% [5, 19]. Therefore, it is important to decide the initiating gonadotropin dosage to avoid OHSS. The goal of the CC combination protocol using a low dose of initiating gonadotropin is to maximize the advantages of CC administration. Although CC milder stimulation has the theoretical advantages of a low risk of OHSS, less gonadotropin consumption, and avoiding the resource wastage of cryopreserving more embryos, but in this trial, only 0.91% of patients had OHSS, with no difference between the two groups. This is due to multiple preventive treatments used in this trial: 150–225 IU HMG initiation, a co-trigger using GnRHa and low-dose hCG and a freeze-only strategy. The current data indicate that CC made it possible to reduce the gonadotropin initiating dose in the PPOS protocol in PCOS women, which is helpful to establish a new, milder stimulation regimen with CC and yields an acceptable pregnancy outcome with the benefit of lower gonadotropin dosage.

It is worth noting that most PCOS women in China have a relatively low BMI. The proportion of higher-BMI (> 25 kg/m^2^) women among PCOS patients is approximately one third. Previous studies reported an association between obesity and an increased gonadotropin requirement [20, 21], so we must be cautious about generalizing our conclusions, especially on the choice of the HMG initiating dose for obese PCOS women. This trial had a relative small sample size, with insufficient power to compare the live birth rate and incidence of OHSS, so a large-sample, prospective randomized controlled trial using milder PPOS with CC is needed to further confirm our conclusions.

## Conclusions

This retrospective cohort trial showed that the milder PPOS protocol with CC in PCOS women led to lower oocyte yields and suboptimal pregnancy outcomes compared to the standard PPOS protocol. The two regimens both achieved a low incidence of OHSS. Milder PPOS with the CC combination regimen showed a higher cancellation rate in exchange for low gonadotropin consumption, but the proportion of viable embryos per oocyte and the implantation rate were higher. Our findings from this CC combination regimen provide a new insight for developing a more patient-friendly protocol for PCOS women.

## Abbreviations

AFC: Antral follicle count
CC: Clomiphene citrate
FET: Frozen embryo transfer
FSH: Follicle-stimulating hormone
ICSI: Intracytoplasmic sperm injection
IVF: In vitro fertilization
LH: Luteinizing hormone
MPA: Medroxyprogesterone acetate
OHSS: Ovarian hyperstimulation
P: Progestin
PCOS: Polycystic ovarian syndrome
PPOS: Progestin-primed ovarian stimulation
